# Motor planning under temporal uncertainty is suboptimal when the gain function is asymmetric

**DOI:** 10.3389/fncom.2015.00088

**Published:** 2015-07-15

**Authors:** Keiji Ota, Masahiro Shinya, Kazutoshi Kudo

**Affiliations:** ^1^Laboratory of Sports Sciences, Department of Life Sciences, Graduate School of Arts and Sciences, The University of TokyoTokyo, Japan; ^2^Research Fellow of Japan Society for the Promotion of ScienceTokyo, Japan

**Keywords:** decision making, risk-sensitivity, Bayesian decision model, response variance, coincident timing task

## Abstract

For optimal action planning, the gain/loss associated with actions and the variability in motor output should both be considered. A number of studies make conflicting claims about the optimality of human action planning but cannot be reconciled due to their use of different movements and gain/loss functions. The disagreement is possibly because of differences in the experimental design and differences in the energetic cost of participant motor effort. We used a coincident timing task, which requires decision making with constant energetic cost, to test the optimality of participant's timing strategies under four configurations of the gain function. We compared participant strategies to an optimal timing strategy calculated from a Bayesian model that maximizes the expected gain. We found suboptimal timing strategies under two configurations of the gain function characterized by asymmetry, in which higher gain is associated with higher risk of zero gain. Participants showed a risk-seeking strategy by responding closer than optimal to the time of onset/offset of zero gain. Meanwhile, there was good agreement of the model with actual performance under two configurations of the gain function characterized by symmetry. Our findings show that human ability to make decisions that must reflect uncertainty in one's own motor output has limits that depend on the configuration of the gain function.

## Introduction

In highly skilled movement, especially in sports, decision making is important for superior performance. For example, a tennis player requires a spatial action plan about where in a court they should aim; a ski jumper requires a temporal action plan about when they should take off. An executed action is associated with a gain/loss. In ski jumping, the take-off jump should be as close to the edge of the ramp as possible to get the best jump length, while take-off too early or too late decreases jump length (Müller, 2009). However, in whatever action they plan, an executed action is not always equal to the planned one because of motor variability (Schmidt et al., 1979; Kudo et al., 2000; van Beers et al., 2004). Thus, both gain/loss associated with action and uncertainty in motor output should be considered for better decision making.

The mathematical method for selecting an optimal plan under conditions of limited uncertainty is known as statistical decision theory (Berger, 1985; Maloney and Zhang, 2010). In particular, Bayesian decision theory, which is a part of statistical decision theory, is a method for optimizing the expected gain/loss. The expected gain/loss is calculated by integrating the gain/loss function assigned to a certain action over a probability distribution of an executed action given a planned action. The Bayesian decision maker plans the action that optimizes the expected gain/loss for any combination of gain/loss function and motor variability (Hudson et al., 2012).

Previous motor control studies have evaluated the optimality of human motor decision making by comparing Bayesian ideal performance with actual human performance (Trommershäuser et al., 2003a,b, 2005; Wu et al., 2006; Hudson et al., 2012; O'Brien and Ahmed, 2013). Some reports have concluded that humans can plan actions that are optimal when considering their own motor variability (Trommershäuser et al., 2003a,b, 2005; Hudson et al., 2012), while other reports have concluded that humans cannot compute the movement strategy that maximizes the expected gain in the presence of such variability (Wu et al., 2006; O'Brien and Ahmed, 2013). Thus, there is inconsistency in published claims concerning the optimality of human action planning.

Two possible factors,—differences in experimental design, and differences in energetic cost—could account for this inconsistency. First, previous studies have reported optimal or suboptimal action plans with different movements and different configurations of the gain function. For example, Trommershäuser et al. (2003a, b, 2005) and Hudson et al. (2012) have demonstrated optimality in a pointing plan under a gain function in which the magnitude of gain/loss remains constant. In contrast, O'Brien and Ahmed (2013) have shown suboptimal reaching and whole-body movement strategies under an asymmetric gain function in which seeking higher values of gain brings participants closer to scoring zero gain (“falling over the cliff”). Therefore, we cannot directly evaluate the relationship between the optimality of the action plan and the configuration of the gain function because the experimental designs among previous studies differed.

Second, previous studies have mainly treated pointing or reaching movement as executed action (Trommershäuser et al., 2003a,b, 2005; Wu et al., 2006; Hudson et al., 2012; O'Brien and Ahmed, 2013). In reaching and pointing movements, energetic cost is proportionally larger as the distance of the required movement is made longer. Because large energetic cost requires participant large effort, energetic cost could be a factor disturbing the measured optimality of action strategies. For example, Hudson et al. (2012) have reported that a discrepancy between ideal and actual performance emerged when optimal but large-cost movements were required during obstacle avoidance (i.e., large excursions).

Here, we used a coincident timing task requiring decision making and compared the Bayesian ideal performance with actual human performance under four different configurations of gain function including those used O'Brien and Ahmed (2013). In the coincident timing task, energetic cost is constant because the participant just presses a button whatever strategy he/she selects. Thus, we can directly evaluate the relationship between the optimality of action plans and the configuration of the gain function excluding the factor of energetic cost as a possible reason for any discrepancy found. We, in fact, found good agreement between the ideal timing strategy and the actual strategy under a symmetric configuration. However, a discrepancy was found under asymmetric configurations. We will discuss possible explanations for this discrepancy. We also observed that larger trial-by-trial compensation occurred following miss trials than after success trials even though the experienced response errors were of the same magnitude.

## Methods

### Participants

Thirty-seven healthy right-handed adults participated in the experiment. Sixteen participants (10 male, 6 female; mean age 28.1 ± 7.8 years) performed Experiment 1, twelve (10 male, 2 female; mean age 22.8 ± 2.8 years) participants performed Experiment 2, and the remaining nine participants (7 male, 2 female; mean age 21.3 ± 2.2 years) performed Experiment 3. All participants were unaware of the purpose of the experiment. This study was approved by the Ethics Committee of the Graduate School of Arts and Sciences, the University of Tokyo.

### Experimental task

**Figure 1:**
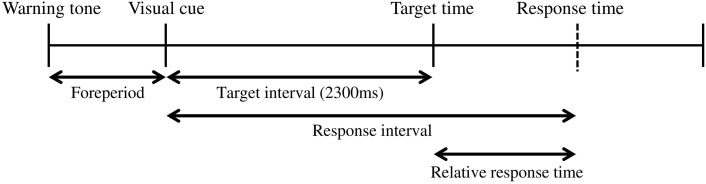
**The coincident timing task**. First, a warning tone is given. After a foreperiod of random duration, a visual cue is then presented. The participant is required to press a button after the visual cue. The relative response time (the difference between the response interval and the target interval) is given to the participant as feedback after every trial.

Figure 1 shows the time sequence of our basic experimental task. First, a warning tone was presented to ready the participants for an upcoming trial. Then, a visual cue was presented on a computer screen as a starting signal (14 inches, 1600 × 900 pixels, refresh frequency 60 Hz; Latitude E5420, Dell, Round Rock, TX, USA). The participants were instructed to press a button after presentation of the visual cue. The response time was recorded as the button-press time relative to the onset time of the visual cue. In each trial, the participants gained a point based on “gain function,” a function that translated the response time to a certain number of points. The details of the gain function are explained in the following section. The foreperiod (interval between the warning tone and the visual cue) was randomly varied between 800 and 1200 ms in steps of 100 ms. The target time was 2300 ms after the visual cue and was fixed throughout the experiment. In our experiment, the target time was associated with gaining 100 points but was not necessarily the time when the participants should respond (see below). The inter-trial interval was 2000 ms. All computerized events were controlled by a program written with LabVIEW software (National Instruments, 2011 Service Pack 1, Austin, TX, USA).

### Experimental condition and procedure

**Figure 2:**
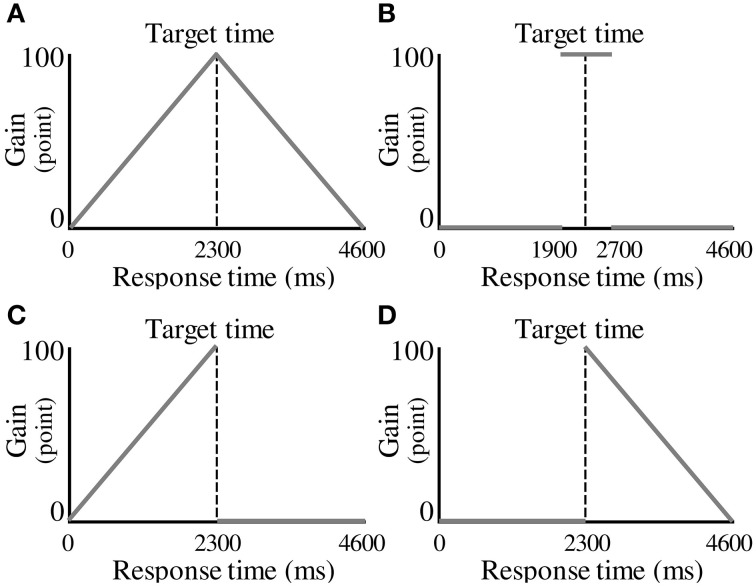
**Experimental conditions. (A)** A symmetric gain function in the No Risk condition. A gain for a trial is determined by the magnitude of the response time. **(B)** A symmetric gain function in the Step condition. A gain of 100 points is given if the participant responds within 400 ms of the target time. **(C)** An asymmetric gain function in the Risk_after_ condition. A gain for a trial is not given (i.e., zero points earned), if the participant responds after the target time. **(D)** An asymmetric gain function in the Risk_before_ condition. The gain function is mirror-imaged across the target response time compared with the Risk_after_ condition.

In each experimental condition, the participants were required to make a decision about when to press a button to maximize the total gain in 100 trials. The gain for a trial was a function of response time, termed the “gain function.” Four conditions were tested, corresponding to different gain functions. The first was characterized as the No Risk condition, which employed a symmetric gain function (Figure 2A). In this condition, a gain for a trial (*G*) was determined from the following equation.

(1) $$G(t)=\{\begin{array}{r} \frac{1}{23}t,ift≦2300\\ -\frac{1}{23}t+200,\;if\;t>2300 \end{array}$$

In the above equation, *t* represents a response time in milliseconds. When the participants responded earlier than the target time, they received a number of points that was a positive linear function of response interval. When the participants responded later than the target time, they received a number of points that was a negative linear function of response interval. A maximum-possible one-trial gain of 100 points could be obtained by responding exactly at the target time.

The second condition was the Step condition, which also had a symmetric gain function (Figure 2B). In the Step condition, a period of constant gain was on the edge of risk of zero gain both at its start and at its termination as represented by the following equation.

(2) $$G(t)=\{\begin{array}{rrr} 0, & if & t\;<1900\\ 100, & if & 1900≦t≦2700\\ 0, & if & t\;>2700 \end{array}$$

The participants received 100 points if they responded within ±400 ms of the target time. However, zero points were given if they responded at less than target −400 ms or at more than target +400 ms. We termed these eventualities “miss trials” and when they occurred, the participants were cautioned by an unpleasant alarm and a flashing red lamp on the screen. The volume of the alarm was 71.5 ± 0.4 dB.

The third condition was characterized as the Risk_after_ condition, which employed an asymmetric gain function (Figure 2C). In the Risk_after_ condition, single-trial gain rose linearly as the target time approached, then plunged to zero after the target time and then remained zero thereafter as represented by the following equation.

(3) $$G(t)=\{\begin{array}{rrr} \frac{1}{23}t, & if & t≦2300\\ 0, & if & t\;>2300 \end{array}$$

Earlier than the target time, the gain function looks the same as that in the No Risk condition. However, zero points were given if the participants responded after the target time. If they missed, they received the same penalty as in the Step condition. This configuration has been used in O'Brien and Ahmed (2013).

The last condition was the Risk_before_ condition in which the gain function of the Risk_after_ condition is mirror-imaged across the target time (Figure 2D). The gain for a trial was determined from the following equation.

(4) $$G(t)=\{\begin{array}{rrr} 0, & if & t<2300\\ -\frac{1}{23}t\;+200, & if & t≧2300 \end{array}$$

In contrast to the Risk_after_ condition, zero points were given if the participants responded before the target time in the Risk_before_ condition. Again, if they missed, they received the same penalty as in the Step condition. For trial-by-trial compensation analysis described below, we defined miss trials as trials when the participants received zero points, success trials as any other trials in all four conditions. Of note, all the trials resulted in success in the No Risk condition because the range in success trials was enough large (i.e., it was 4600 ms).

In each trial, we provided the participants with feedback information consisting of the relative response time calculated by response time–the target time, the gain for the trial, and the cumulative total gain. All the participants performed 10 trials for practice. This practice session was conducted to give them a feel for the length of time from the visual cue to the target time. In this session, we only provided them with the relative response time (i.e., no gain function was applied). After the practice session, all the participants performed 100 trials in the No Risk condition as a first experimental session. In the second experimental session, the participants who were assigned to Experiment 1 performed 100 trials in the Risk_after_ condition. The participants who were assigned to Experiment 2 performed 100 trials in the Risk_before_ condition. Those who were assigned to Experiment 3 performed 100 trials in the Step condition. Each condition (No Risk and Risk_before_/Risk_before_/Step) was conducted as separate experimental session. The participants rested for several tens of seconds between sessions.

Before running each session, we explained the structure of the gain function with a figure visualizing it (see Supplementary Figure 1). Thus, the structure of the gain function was known to the participants before testing. In the figure, following information was also included: 100 points could be gained when the relative response time was 0 ms in the No Risk, Risk_after_, and the Risk_before_ conditions, and when the relative response time was within ±400 ms of the target in the Step condition. However, the length of time from the visual cue to the target time was not described; thus, the participants did not know that it was 2300 ms. Also, before performing the No Risk condition, the participants did not know the gain structure that would be used in the next condition.

We instructed them to maximize total gain in each condition. Thus, they were required to make a decision about when to press a button to maximize the total gain. Actual monetary rewards/penalties were not used (see limitation related to this experimental procedure).

### Model assumptions

We calculated the ideal strategy that maximizes the expected gain by a Bayesian decision-theoretic approach for each participant and for all conditions (Hudson et al., 2012). Our model consisted of two sets and two functions. The two sets were: possible response strategy *T* (motor decision), and executed response time *t* (motor output). The two functions were: probability distribution of executed response *P*(*t*|*T*), and gain function *Gt*). Given a particular planned strategy, a particular response is stochastically executed. This is considered the uncertainty in motor output. In this study, we assumed that the produced response time *t* is distributed around the planned response time *T* according a Gaussian distribution (see Supplementary Table 1) as follows.

(5) $$P\;(t|T)\;=\frac{1}{\sqrt{2\pi \sigma^{2}}}exp\left[-\frac{{\left(t-T\right)}^{2}}{2\sigma^{2}}\right]$$

Then, given execution of a particular response time, the gain is given according to the gain function *G*(*t*). Given both *P*(*t*|*T*) and *G*(*t*), the expected gain *EG*(*T*) as a function of planned response time *T* is calculated by the following equation.

(6) $$EG(T)=\int_{-\;\infty }^{\infty }G(t)\cdot P(t|T)dt$$

Once we had measured the response variance σ for each participant and condition, we could calculate the optimal mean response time T^*^ by maximizing Equation (6). A Bayesian decision maker chooses a response time T^*^ for any given gain function *G* (*t*) and response variance σ. This can be regarded as a theoretical risk-neutral optimal response.

### Estimation of 95% confidence interval of optimal response time

Furthermore, we estimated the 95% confidence interval of the optimal mean response time T^*^ by bootstrapping (3000 resamples) for the Risk_after_ and the Risk_before_ conditions. We then examined whether the actual response time is within this 95% confidence interval in the Risk_after_ condition and in the Risk_before_ condition. In a range from σ = 0 to 0.4 in steps of σ = 0.001, we first calculated each optimal mean response time *X*_*opt*1_(σ_0_), *X*_*opt*2_(σ_0.001_), …, *X*_*opt*400_ (σ_0.4_) by maximizing Equation (6). Here focusing on *X*_*opt*1_(σ_0_), we simulated 100 trials of a task execution responding by this optimal mean response time and having this response variance (in this case *X*_*opt*_*1* = 2300, σ = 0) using a MATLAB *randn* function. We repeated this process 3000 times and obtained bootstrap samples *x*_1_ = (*x*_1*t*1_, *x*_1**t**2_, …, *x*_1*t*100_), *x*_2_ = (*x*_2*t*1_, *x*_2*t*2_, …, *x*_2*t*100_), …, *x*_3000_ = (*x*_3000_*t*_1_, *x*_3000_*t*_2_, … *x*_3000*t*100_). For each bootstrap sample *x*_*b*_ (*b* = 1, 2, …, 3000), we calculated the average value of its samples ${\hat{\mu }}_{b}=\;\frac{1}{100}\cdot \sum_{i=1}^{100}\sum_{}^{}\left(x_{bti}\right).$. After sorting these average samples ${\hat{\mu }}_{b}(b\;=\text{ 1},\;2,\cdot \cdot \cdot ,\;3000)$ in ascending order, we defined a 2.5% and a 97.5% point in these samples as the 95% confidence interval in optimal mean response time T^*^. By repeating the above processes from *X*_*opt*1_(σ_0_) to *X*_*opt*400_(σ_0.4_), we estimated the 95% confidence intervals of each optimal mean response time.

If the observed mean response times were within these 95% confidence intervals, we would conclude that the participant plans optimal and risk-neutral timing strategies. In the Risk_after_ condition, observed times longer than the confidence intervals would indicate suboptimal and risk-seeking strategies. Observed times shorter than the confidence intervals would indicate suboptimal and risk-averse strategies.

### Optimal response times calculated from the measured distributions

**Figure 3:**
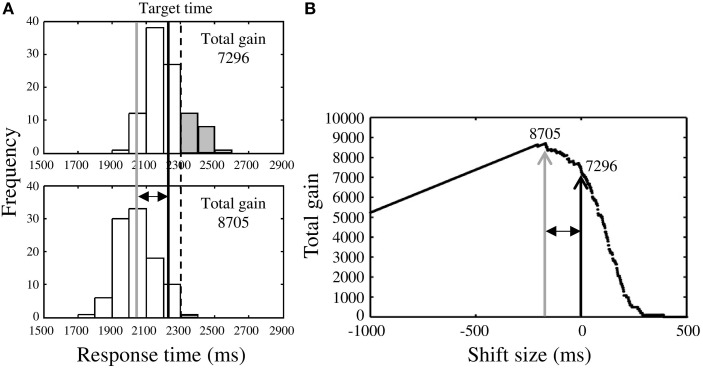
**Procedure for estimating optimal response time. (A)** Upper panel shows the response distribution obtained in the Risk_after_ condition. The black solid line indicates the observed mean response time and the dotted line is the target time. Lower panel shows the distribution after shifting along the time axis to maximize total gain. The gray solid line indicates the optimal mean response time estimated in this way. In this case, the observed mean response time is found to locate closer to the target time than optimal time (risk-seeking behavior), thus the total gain was reduced by some miss trials (gray bars). **(B)** Black arrow indicates the total gain (7296 points) when the distribution in the Risk_after_ condition is not shifted. The distribution was shifted until the highest total gain (8705 points; gray arrow) was obtained. This optimal shift size is represented by the two-headed arrow. The estimated optimal mean response time (gray solid line in **A**) is the sum of the observed mean response time and the optimal shift size. Although two distributions shown in **(A)** are identical, these distributions do not have the same shape because the sample sizes included in one bin are different between distribution in upper panel and that in lower panel.

Although we had confirmed that the response distributions were Gaussian (see Supplementary Table 1), we also calculated optimal mean response time for the Risk_after_ and the Risk_before_ conditions using the measured response distributions. In the Risk_after_ condition (Figure 3A, upper panel), once we had obtained the response distribution we simply shifted it back and forth to identify the maximal total gain for that distribution (Figure 3A, lower panel). In Figure 3A, we show the case of shifting the measured distribution back. We defined the optimal mean response time as the mean response time of the optimized distribution (gray solid line in Figure 3A). The estimated optimal mean response time was always earlier than the target time in the Risk_after_ condition. The difference between the estimated optimal response time and the target time reflected each participant's own variance in response time, (i.e., the larger one's variance, the earlier the optimal response time, and vice-versa. This effect is visualized in **Figure 5** as the solid curve).

In Figure 3B, the black arrow indicates the total gain when the measured distribution is not shifted and the gray arrow indicates the highest total gain possible for that distribution under shifting. The two-headed arrows in Figures 3A,B represent the optimal shift size.

Finally, we compared the estimated optimal mean response times with the observed mean response times (black solid line in Figure 3A). In this case, the observed time was closer to the target time than optimal, indicating risk-seeking. We also applied this approach to the Risk_before_ condition. We compared the mean, as opposed to median optimal and observed times because the measured distributions were Gaussian.

### Trial-By-trial compensation strategy

In addition to determining the response time strategies based on all trials, we examined compensation against the most recent response error based on a trial-by-trial analysis. These results were then compared between/within the No Risk and the Risk_after_/Risk_before_ conditions. The magnitude of the response error experienced on the current trial is known to influence the response in the following trial (Thoroughman and Shadmehr, 2000; Scheidt et al., 2001). Thus, the compensation size in the following trial can be proportional to the current magnitude of response error. Additionally, it has been shown that humans adjust future motor behavior according to rewarding and non-rewarding outcomes experienced (Wrase et al., 2007). Therefore, in addition to the compensation strategy against response error, we hypothesized that the compensation size following miss trials would be larger in the Risk_after_/Risk_before_ conditions than the compensation size following success trials in the No Risk condition.

We defined the compensation on the current trial, trial *n*, by subtracting the response time on the current trial, from that on the following trial, trial *n* + 1, as in Equation (7).

(7) $$Compensation_{n}\;=RT_{n\;+\;1}-RT_{n}$$

We supposed that the compensation occurs around mean response time in both conditions, thus we defined response error as response time–mean response time in this analysis. The compensation size was anticipated to depend on the magnitude of response error (in other words, the magnitude of deviation between the current response time and mean response time).

**Figure 4:**
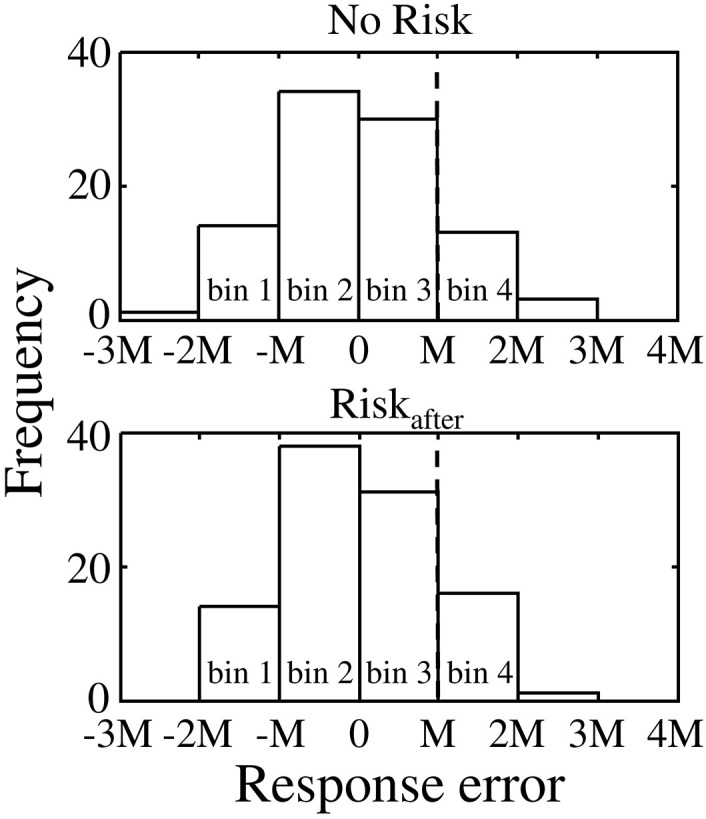
**Procedure of trial by trial analysis**. Upper panel shows the response error distribution obtained in the No Risk condition. Lower panel shows the response error distribution obtained in the Risk_after_ condition. The value of “0” in the x-axis corresponds to mean response error in both conditions. For comparable analysis between conditions, we sorted trials into four bins separated by the range “*M*.” *“M”* indicates the absolute value of the difference between mean response time in the Risk_after_/Risk_before_ condition and the target time. It allows data from different participants to be combined. The average value of *“M”* across the participants was 153.8 ± 42.4 in Experiment 1 and 194.5 ± 40.5 in Experiment 2. In this example, bin 4 (i.e., the range: M< *error*_*n*_ ≤ 2M) was the area of miss trial in the Risk_after_ condition. In contrast, the same bin was the area of the success trial in the No Risk condition. We especially focused on the compensation size following to errors included in these ranges.

To compare the compensation size on the following to miss/success trials, we defined the absolute value of the difference between mean response time in the Risk_after_/Risk_before_ condition and the target time as “*M*” for convenience, and sorted trials into following four bins, −2M < *error*_*n*_ ≤ −M (bin 1), −M < *error*_*n*_ ≤ 0 (bin 2), 0 < *error*_*n*_ ≤ M (bin 3), and M < *error*_*n*_ ≤ 2M (bin 4) for Experiment 1 and −2M ≤ *error*_*n*_ < −M (bin 1), −M ≤ *error*_*n*_ < 0 (bin 2), 0 ≤ *error*_*n*_ <M (bin 3), and M≤ *error*_*n*_ < 2M (bin 4) for Experiment 2. Figure 4 shows the error distributions separated by the bins. With these procedures, we can evaluate the compensation sizes based on same magnitude of response error between conditions. Scaling by “*M*” also allows data from different participants to be combined. The last bin in Experiment 1 and the first bin in Experiment 2 are in the areas that result in miss trials in the Risk_after_/Risk_before_ conditions. Errors −2M or less and more than 2M in Experiment 1 and errors less than −2M and 2M or more in Experiment 2 were excluded from the analysis because only small numbers of trials were obtained within these ranges. We collected errors on trial *n* in each bin and calculated the average compensation size in each bin. Repeating this procedure for each participant and condition, we compared the average compensation size across the participants against the magnitude of response error between/within conditions for each bin. Because the distributions of the compensation sizes across the participants were Gaussian (see Supplementary Table 2), we calculated the average value.

### Statistical analysis

We conducted paired *t*-tests to examine the significance of differences between optimal and observed values for response time and total gain in all conditions. We also conducted two-way repeated-measures ANOVA to determine differences in trial-by-trial compensation strategy between the No Risk condition and the Risk_after_/Risk_before_ conditions. A *p* < 0.05 was regarded as statistically significant. Cohen's *d* measure for the *t*-test was calculated to determine the magnitude of mean differences.

Trials with response times more than ±2.5 standard deviations from the mean were excluded from the analysis as outliers. Average number of trials excluded across the participants and two conditions was 2.4 ± 1.5 in Experiment 1, 2.3 ± 1.3 in Experiment 2, and 1.7 ± 0.7 in Experiment 3.

## Results

### Discrepancies with optimal strategy

**Figure 5:**
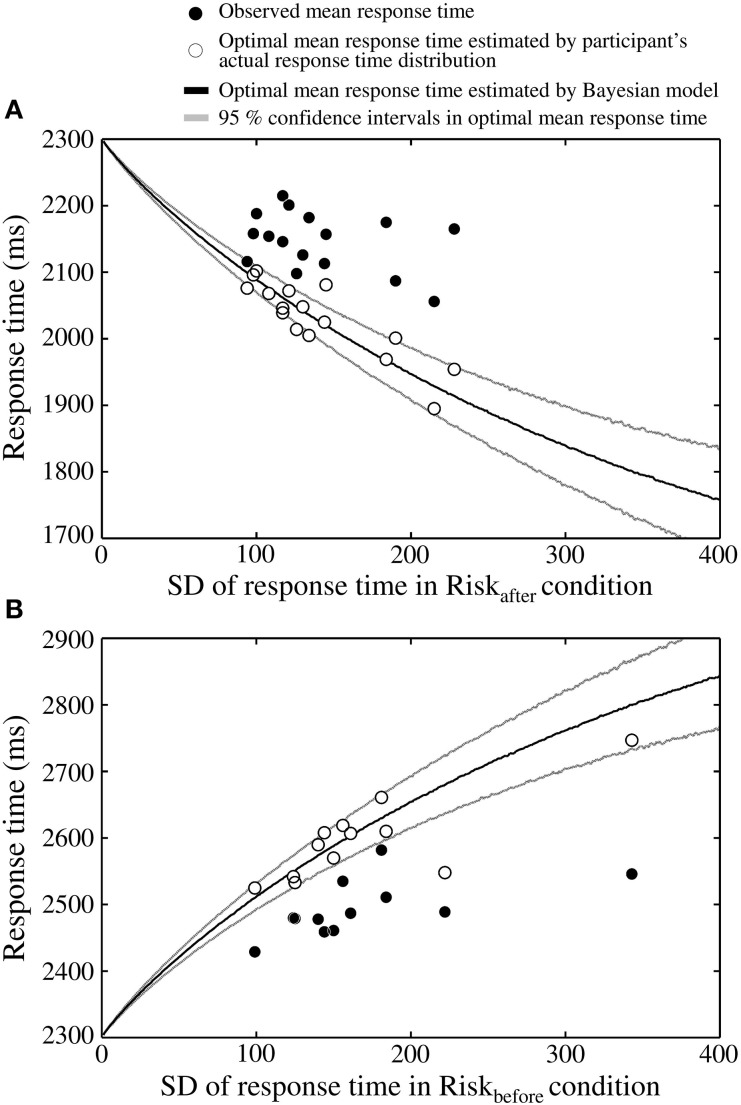
**The participants adopt a risk-seeking strategy. (A)** Results in the Risk_after_ condition, one dot of each color corresponds to a participant. Theoretically, the optimal mean response time must be shorter than the target time as an increasing function of the variability of one's response time (the *x*-axis shows the SD of the response time in the Risk_after_ condition as an index of this variability). However, the observed mean response times (filled circles) are closer to the target time than optimal. Thus, a discrepancy is seen between actual strategy and the ideal strategy. Open circles indicate optimal mean response times estimated by shifting the participant's actual response time distributions. Black curve indicates the optimal mean response time estimated by a Bayesian model (Equation 6). Gray curves indicate the 95% confidence intervals of the optimal mean response times, which were calculated by 3000 replications of a bootstrap algorithm. The optimal response times estimated from the actual distributions locates roughly within the 95% confidence intervals. **(B)** Results in the Risk_before_ condition. The same risk-seeking behavioral tendency is observed. In this condition, the optimal mean response time must be longer than the target time as an increasing function of response variability. The observed response times are again closer than optimal to the target time.

We found discrepancies between the Bayesian ideal strategy and the actual human strategy in the Risk_after_ and the Risk_before_ conditions. The observed mean response times and the optimal mean response times calculated from the measured distributions were plotted against the standard deviation (SD) of response time in the Risk_after_ condition for all 16 participants (Figure 5A). The optimal mean response times calculated by the Bayesian model and their 95% confidence intervals were also plotted. As shown in Figure 5A, the optimal mean response time moves further from the target time as response variance increases. However, for all participants, except one, the observed mean response time was closer to the target time than the optimal mean response time calculated either from the Bayesian-theoretical 95% confidence interval or from the measured distribution. This result suggests that the participants took higher-than-optimal risks given their own variance in response time, which is classified as a suboptimally risk-seeking tendency.

The participants were also suboptimal in the Risk_before_ condition in the sense that they were in general faster to respond than predicted by the optimal model (Figure 5B). In the Risk_before_ condition, the optimal time is later than the target time. We found that all 12 participants responded closer to the target time than the Bayesian-theoretical 95% confidence interval for the optimal mean response time and the optimal response time calculated from the measured distribution. Therefore, a risk-seeking strategy was shown under an asymmetric gain function regardless of the location of the penalty region.

We also found that the SD of the response time was significantly correlated with that of the observed mean response time in the Risk_before_ condition (*r* = 0.59, *p* < 0.05; but was not significantly correlated in the Risk_after_ condition, *r* = −0.36, *p* = 0.17). Thus, in the Risk_before_ condition, participants who had large response variance responded further to the target time than those who had small variance. This result raises a possibility that our participants might have chosen response times reflecting the size of their own response variance. However, their timing strategy was not optimal from the perspective of Bayesian decision theory.

### The effect of the symmetry of the gain function

Actual human strategy agreed with the Bayesian ideal strategy under a symmetric gain function. We compared the optimal mean response time estimated by the Bayesian model with the observed mean response time in all four conditions (Table 1). Paired *t*-tests showed that across participants, the optimal mean response time was not significantly different from that observed in the No Risk [*t*_(36)_ = −0.08, *p* = 0.94, *d* = −0.02] and the Step conditions [*t*_(8)_ = 1.76, *p* = 0.12, *d* = 0.87]. However, the observed mean response time in the Risk_after_ condition was significantly longer than the optimal mean response time [*t*_(15)_ = −8.00, *p* < 0.001, *d* = −2.35], and was significantly shorter in the Risk_before_ condition [*t*_(11)_ = 6.68, *p* < 0.001, *d* = 2.04]. Thus, the participants planned optimal timing strategies only under symmetric gain functions.

**Table 1:** **Suboptimal strategies are adopted under asymmetric gain functions**.

	**Condition**	***N***	**Optimal response time (ms)**	**Observed response time (ms)**	**Effect size (d)**	**Optimal total gain (point)**	**Observed total gain (point)**	**Effect size (d)**
Symmetry	No risk	37	2300.0±0.0	2300.9±72.9	−0.02	9104.0±216.0	9070.6±245.8^*^	0.14
	Step	9	2300.0±0.0	2285.3±23.9	0.87	9585.6±158.4	9600.0±194.4	−0.08
Asymmetry	Risk_after_	16	2029.5±56.1	2146.2±42.4^***^	−2.35	8382.7±347.8	7686.8±501.3^***^	1.61
	Risk_before_	12	2611.8±69.9	2495.2±40.3^***^	2.04	8160.6±442.3	7833.8±556.9^***^	0.65

Optimal response time and optimal total gain were calculated with a Bayesian model (Equation 6). There is no significant difference between observed and optimal response times in the No Risk and Step conditions (symmetric gain functions), but there is a significant difference in the Risk_after_ and Risk_before_ conditions (asymmetric gain functions). All data shown are averages across the participants ± standard deviation of the mean.

* indicates *p* < 0.05 and

*** *indicates *p* < 0.001*.

Looking at total gain, the average value of the observed total gain across participants was not significantly different from that of the optimal total gain in the Step condition [*t*_(8)_ = −0.33, *p* = 0.75., ^*^d = −0.08]. The average observed total gain was significantly smaller than the average optimal gain in the No Risk [*t*_(36)_ = 2.38, *p* < 0.05, *d* = 0.14], the Risk_after_ [*t*_(15)_ = 7.14, *p* < 0.001, *d* = 1.61], and the Risk_before_ [*t*_(11)_ = 6.68, *p* < 0.001, *d* = 0.65] conditions. Although the total gain was significantly smaller than the optimal gain in the symmetric No Risk condition, its effect size was apparently small compared with the asymmetric Risk_after_ and Risk_before_ conditions. Taken together, we confirm that an optimal strategy for maximizing expected gain could be computed under a symmetric gain function, but not under an asymmetric gain function.

### Learning effects on timing strategy

We analyzed timing strategy on a whole block of 100 trials and showed its suboptimality in the Risk_after_ and the Risk_before_ conditions. However, there is a possibility that the participants gradually learned the strategy thorough trials. To investigate this possibility, we compared the mean response time over the first 50 trials with the mean over the last 50 trials across participants. Paired *t*-tests showed that early and late mean response times were not significantly different in the Risk_after_ condition [*t*_(15)_ = 1.48, *p* = 0.16] and the Risk_before_ condition [*t*_(11)_ = 0.53, *p* = 0.61]. Furthermore, we conducted paired *t*-tests in each participant excluding trials that were classified as outliers. The results showed that early and late mean response times were significantly different for only 1 out of 16 participants in the Risk_after_ condition [*t*_(47)_ = 2.10, *p* < 0.05 for P13], and for 2 out of 12 participants in the Risk_before_ condition [*t*_(47)_ = −2.76, *p* < 0.01 for P18; *t*_(47)_ = 2.68, *p* < 0.01 for P22]. Therefore, we concluded that participants did not learn timing strategy through trials. This result is consistent with previous studies claiming no evidence of learning effects on movement plans (Trommershäuser et al., 2003b, 2005; Wu et al., 2006; O'Brien and Ahmed, 2013).

### Trial-by-trial compensation strategy

**Figure 6:**
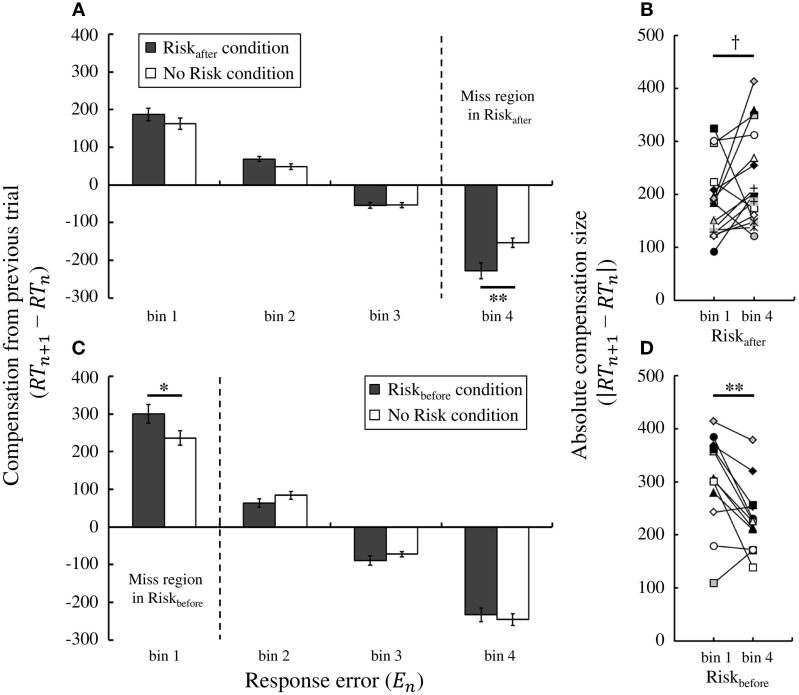
**Trial-by-trial compensation strategy adopted by the participants. (A)** Result of Experiment 1. Compensation based on feedback from the previous trial depends on the magnitude of the experienced response error. The average compensation across participants is plotted against the magnitude of response error for the Risk_after_ and No Risk conditions. A simple main effect test reveals that the average compensation in the Risk_after_ condition is to significantly shorter times than that in the No Risk condition in bin 4 (penalty region in the Risk_after_ condition), which indicates that participants overcompensate following miss trials compared with success trials in which the magnitude of the response error is held the same. **(B)** Individual data in the Risk_after_ condition, each colored symbol corresponds to a participant. Overcompensation also occurred following miss trials compared with success trials within the same Risk_after_ condition. Absolute value of the average compensation size across the participants in bin 4 was marginally significantly larger than that in bin 1. **(C)** Result of Experiment 2. The same tendency is observed in the Risk_before_ condition. The average compensation in the Risk_before_ condition is to significantly longer times than that in the No Risk condition in bin 1 (penalty region in the Risk_before_ condition). **(D)** Individual data in the Risk_before_ condition. The absolute compensation size in bin 1 was significantly larger than that in bin4. ^*^^†^ indicates *p* < 0.10, ^*^ indicates *p* < 0.05, and ^**^ indicates *p* < 0.01. Error bars indicate standard error of the mean. Individual data for the compensation size in all bins is shown in Supplementary Figure 3.

We then compared trial-by-trial compensation strategies between the No Risk and the Risk_after_/Risk_before_ conditions. To this end, in Figure 6A we plotted the average compensation size across participants against the magnitude of response error on the previous trial in Experiment 1. We performed two-way repeated-measures ANOVA on the compensation size. The levels were condition (2: Risk_after_ condition and No Risk condition) and bin (4: bin 1–4). We found a main effect of bin [^*^F_(1.63, 24.46)_ = 185.02, *p* < 0.001]. Thus, the average compensation size changed based on the experienced response error. Furthermore, we also found an interaction effect [^*^F_(2.23, 33.52)_ = 6.36, *p* < 0.01] and a simple main effect of condition in bin 4 [*F*_(1, 15)_ = 10.78, *p* < 0.01], but not in any other bins [^*^Fs_(1,15)_ < 4.16, *ps* > 0.05]. Of note, miss trials in the Risk_after_ condition are included in bin 4 (i.e., the range: M < error_n_ ≤ 2M). Moreover, in bin 4, the average compensation size in the Risk_after_ condition was significantly larger than that in the No Risk condition [*t*_(15)_ = −3.28, *p* < 0.01, ^*^d = −1.03]. These results suggest that the participants used statistically the same compensation strategy following success trials (bins 1–3 in both conditions), but compensated more strongly following miss trials (bin 4 in the Risk_after_ condition) compared with success trials (bin 4 in the No Risk condition), even though response errors in both bins were of the same magnitude (see Supplementary Figure 2).

Larger compensation after misses was also seen in Experiment 2 (Figure 6C). Again, we performed two-way repeated-measures ANOVA on the compensation size. We found a main effect of bin [^*^F_(1.59, 17.48)_ = 192.30, *p* < 0.001] and an interaction effect [*F*_(3, 33)_ = 4.12, *p* < 0.05]. We also found a simple main effect of condition in bin 1 [*F*_(1, 11)_ = 6.90, *p* < 0.05], but not in any other bins [^*^Fs_(1, 11)_ < 2.70, *ps* > 0.05]. Bin 1 (i.e., the range: −2M ≤ error_n_ < M) is in the area of miss trials in the Risk_before_ condition. Similarly to Experiment 1, the average compensation size in the Risk_before_ condition was larger than that in the No Risk condition in bin 1 [*t*_(11)_ = 2.63, *p* < 0.05, *d* = 0.80]. Therefore, we confirmed larger compensation following miss trials as a robust result regardless of the location of the penalty region.

We also confirmed that larger compensation occurred within the same Risk conditions. Paired *t*-test revealed that the absolute value of the average compensation size in bin 1 was significantly larger than that in bin 4 within the Risk_before_ condition [*t*_(11)_ = 3.45, *p* < 0.01, *d* = 0.91; individual data were shown in Figure 6D]. Within the Risk_after_ condition, the absolute compensation size in bin 4 was marginally significantly larger than that in bin 1 [*t*_(15)_ = −1.78, *p* = 0.096, ^*^d =−0.53; individual data were shown in Figure 6B]. Magnitude of response errors was same in bin 1 and bin 4 but sign of errors was different. Significant difference between bin 1 and bin 4 in the No Risk condition was found neither in Experiment 1 [*t*_(15)_ = 0.75, *p* = 0.47, *d* = 0.18] nor in Experiment 2 [*t*_(11)_ = −0.41, *p* = 0.69, ^*^d = −0.15].

## Discussion

### Summary of results

We directly evaluated the relationship between the optimality of action plans and the configuration of the gain function. With the coincident timing task, we could exclude the energetic cost factor, which might disturb an optimal action plan. Compared with Bayesian optimal timing strategy, our participants planned suboptimal strategies under asymmetric configurations. They tended to respond closer than optimal to times presenting the risk of zero gain. Under symmetric configurations, good agreement between the observed and optimal strategies was found. Furthermore, larger compensation occurred following miss trials compared with success trials even though the experienced response errors were of the same magnitude.

### Suboptimal decision making

We investigated whether humans can calculate an optimal timing strategy that maximizes the expected gain under four configurations of the gain function. In the Step condition, a constant value of gain was on the edge of risk of zero gain. The gain function has a symmetric configuration in this condition. Most of the relevant previous studies have used this type of gain function and have reported optimal movement planning (Trommershäuser et al., 2003a,b, 2005; Hudson et al., 2012). We likewise showed that strategies were optimal in the Step condition. In the Risk_after_ condition, higher values of gain come with higher risk. We found a discrepancy between the ideal Bayesian model and actual strategy in the Risk_after_ condition, participants showing a risk-seeking strategy. Our finding is consistent with a previous study that reports risk-seeking strategy under a similar gain function during reaching and whole-body movement tasks (O'Brien and Ahmed, 2013). In addition to the Risk_after_ condition, we applied the Risk_before_ condition in which the configuration of the Risk_after_ condition was inverted with respect to time. We confirmed a risk-seeking strategy also in the Risk_before_ condition. Therefore, these results suggest that human action plans tend to be suboptimal under situations in which higher values of gain occur closer to zero gain regardless of the location of risk. On the other hand, action plans could be optimal under situations in which a constant value of gain was close to zero gain.

A symmetric gain configuration was applied in the No Risk and Step conditions, while an asymmetric configuration was applied in the Risk_after_ and Risk_before_ conditions. Wu et al. (2006) have investigated the endpoint of pointing movements under both symmetric and asymmetric expected gain landscape. Theoretically, the optimal endpoint in that study lay within the target circle under a symmetric expected gain landscape but lay within the penalty circle and did not cover the target circle under an asymmetric expected gain landscape. These researchers showed that an intuitive strategy to aim within the target circle could be adopted, but a counterintuitive strategy to aim within the penalty circle could not be adopted. Even in our experiment, the participants might not easily detect when they should press a button because optimal response time depends on response variance under an asymmetric configuration of the gain function. Therefore, our findings indicate a limitation on information processing and computational ability in decision making under uncertainty in motor output as well as in economic decision making (Simon, 1956).

### Distortion of subjective value

In the field of behavioral economics, prospect theory (Kahneman and Tversky, 1979) and cumulative prospect theory (Tversky and Kahneman, 1992) claim that irrational decision making is caused by a distortion of probability weighting from the actual probability and a distortion of subjective utility from the actual gain/loss. Prospect theory gives two reasons for risk-seeking behavior.

One reason would be an inappropriate estimation of the participant's own variance in response time (Wu et al., 2009; Nagengast et al., 2011; O'Brien and Ahmed, 2013). Wu et al. (2009) have shown that participants under-weighted small probabilities and over-weighted large probabilities when they made a decision whether to point to a riskier target bar or a safer target bar. O'Brien and Ahmed (2013) have also shown a similar distortion of probability weighting during a reaching task. These reports indicate that our participants might have believed that they had smaller response variability than they actually did. Such an inappropriate estimation of their own variance would have influenced them to approach the penalty zone more closely.

Before performing the Risk_after_/Risk_before_ condition, participants had only experienced 100 trials in the No Risk condition. Thus, they may not have had enough experience with the task to know their own response variance, but the report of Zhang et al. (2013) calls into question the idea that more practice would have helped. These researchers have shown that the distribution of a reaching endpoint was recognized as an isotropic distribution rather than the actual anisotropic distribution, and that this inaccurate estimation persisted even after extensive practice. This report indicates that an inappropriate estimation of one's own variance is not necessarily caused by lack of practice. Thus, the ability to recognize one's own variance in motor output appropriately may have limitations.

The second reason would be inappropriate evaluation of gain/loss (Lee, 2005; O'Brien and Ahmed, 2013). Risk-seeking in decision making arises when the subjective utility of gain is overvalued against the objective value (Lee, 2005). Here, higher values of gain came with a higher risk of zero gain in the Risk_after_/Risk_before_ conditions. O'Brien and Ahmed (2013) showed that most participants overvalued point reward and undervalued point penalty under this type of gain function. This inappropriate evaluation of gain/loss would also influence our participants to respond closer to the point where gains of zero began. However, we could not distinguish which distortion most affected risk-seeking behavior using the above analyses. Thus, our remaining issue is to specify them using other experimental paradigms.

### Trial-by-trial analysis

We also investigated differences in trial-by-trial compensation strategy between/within the No Risk and the Risk_after_/Risk_before_ conditions. We found larger compensations following miss trials compared with success trials between the No Risk and the Risk_after_/Risk_before_ conditions with the same magnitude of response errors (see Supplementary Figure 2). The sign of response errors was same in comparison between the conditions. In comparison within the Risk_after_/Risk_before_ conditions, we also found that larger compensations occurred following miss trials compared with success trials with different sign of response errors. We assume that this is because of motivation to avoid consecutive misses.

Error feedback is necessary for motor adaptation (motor learning). Previous studies have investigated how the magnitude of error influences subsequent adaptation. These studies have reported that the size of the adaptation has a linear relationship with the magnitude of past errors (Thoroughman and Shadmehr, 2000; Scheidt et al., 2001). Linear adaptation against error is an element in minimizing future errors. However, recent studies have shown that motor adaptation does not depend simply on the magnitude of error. A nonlinear relationship has been observed when the subjective value, directional bias, statistical properties, and relevance of errors are experimentally manipulated (Fine and Thoroughman, 2007; Wei and Körding, 2009; Trent and Ahmed, 2013).

In our task, the subjective value of error (Trent and Ahmed, 2013) was different between conditions. Errors over/within the target time were cautioned in the Risk_after_/Risk_before_ condition, while same magnitude of errors was not cautioned in the No Risk condition. Trent and Ahmed (2013) have shown that weaker adaptation and weaker error sensitivity in response to errors further from the penalty region. This suggests that nonlinear adaptation is an effective motor control strategy for avoiding penalty. In our study, larger compensation was observed in response to errors that were recognized as misses. This suggests that the larger compensation strategy is an effective control heuristic for avoiding consecutive misses. This tendency was robust regardless of the location of the penalty region. Thus, our results support the view that compensation on the following trial is influenced not only by the magnitude of the error but also by the subjective value of the error.

### Decision making on the sports field

Risk-seeking behaviors are sometimes seen in real life on the sports field. For example, professional NBA basketball players attempt consecutive three-point shots after they succeed in making a three-point shot even though the probability of further points is decreased (Neiman and Loewenstein, 2011), possibly due to enhanced self-confidence. NBA players are also unwilling to shoot during an early stage of the shot clock even though higher points per possession can be obtained by shooting more frequently (Skinner, 2012). This is possibly because of overconfidence about shot opportunities during later stages (Skinner, 2012). Therefore, suboptimal decision making would have the effect of degrading the performance of beginners as well as of experts in a variety of sports.

Both symmetric and asymmetric gain functions are seen on the sports field. Examples of the former occur in archery, Japanese archery, and shooting. In these sports, a gain is distributed symmetrically around the center of the target. Accuracy in hitting the center of the target is a crucial factor in performance. Examples of the latter gain functions occur in tennis, volleyball, golf, and ski jumping. In tennis, a ball bouncing as close as possible to the line marking the edge of the court would result in scoring a point, while a ball bouncing beyond the line would cost the player a point. In such a situation, appropriate decision making about where to aim as well as the accuracy of the aim are both critical factors. We have shown that humans cannot always make such appropriate decisions that consider variance in motor output. Therefore, the implication of our results for coaches and trainers, especially in sports with asymmetric gain functions is that it is important to fashion a risk-handling strategy optimized for each player's skill level.

### Limitations

In this study, we compared the observed mean response time calculated over 100 trials with the optimal mean response time in the Risk_after_/Risk_before_ condition and in each participant. Taking into account the fact that the location of the optimal mean response times move further from the target time as response variance increases, the observed mean response times were closer than optimal to the target time in both conditions (Figures 5A,B). However, a possibility remains that this observed response time would have approached optimal if the participants had been able to decrease their response variance through more practice. Therefore, our remaining issue is to investigate this possibility with a longitudinal study.

As another limitation, we instructed the participants to maximize the total gain but we did not use an experimental procedure giving them real monetary rewards in accordance with their performance. This raises the possibility that real monetary rewards would have induced risk-neutral behavior. However, it has been shown that real and virtual rewards induced similar performance in economic decision making (Bowman and Turnbull, 2003), autonomic response (skin conductance response) patterns resulted from monetary wins or loses (Carter and Smith Pasqualini, 2004), and brain activation patterns (Miyapuram et al., 2012). Therefore, we consider that use of real monetary rewards would have a small effect on our participant's risk-seeking behavior. However, it would be interesting to investigate motor decision making in situations in which a one-trial decision wins a high-priced award, such as a game-winning shot or a tour-winning putt.

## Author contributions

KO, MS, and KK conceived and designed the experiments. KO performed the experiments. KO and MS programmed the simulations and KO analyzed the results. KO, MS, and KK interpreted the results. KO wrote the manuscript, MS and KK commented and revised the manuscript. KO, MS, and KK approved final version of manuscript.

### Conflict of interest statement

The authors declare that the research was conducted in the absence of any commercial or financial relationships that could be construed as a potential conflict of interest.
