# Supplementary material for: Motor planning under temporal uncertainty is suboptimal when the gain function is asymmetric
Source: Front Comput Neurosci. 2015 Jul 15;9:88. doi: 10.3389/fncom.2015.00088 (PMC4502360; doi:10.3389/fncom.2015.00088)
Supplement: Supplementary file 1 [file DataSheet1.PDF]

Supplementary Material

Motor planning under temporal uncertainty is suboptimal when the gain function is asymmetric

Keiji Ota[1, 2]*, Masahiro Shinya[1], Kazutoshi Kudo[1]*

[1] Department of Life Sciences, Graduate School of Arts and Sciences, The University of Tokyo, Tokyo, Japan.

[2] Research Fellow of Japan Society for the Promotion of Science, Tokyo, Japan.

*** Correspondence:** Keiji Ota, Department of Life Sciences, Graduate School of Arts and Sciences, The University of Tokyo, Komaba 3-8-1, Meguro, Tokyo 153-8902, Japan
Email: keiji.o.22@gmail.com

Kazutoshi Kudo, Department of Life Sciences, Graduate School of Arts and Sciences, The University of Tokyo, Komaba 3-8-1, Meguro, Tokyo 153-8902, Japan
Email: kudo@idaten.c.u-tokyo.ac.jp

1. Supplementary Figures and Tables

1-1. The figures of gain function presented to the participants

Before running each condition, we instructed the participants the structure of gain function with figures describing it (Supplementary Figure 1). In Supplementary Figure 1, horizontal axis shows relative response time which the participants received as feedback information. By presenting these figures, the participants could know the structure of gain function before they started to perform the coincident timing task in each condition.

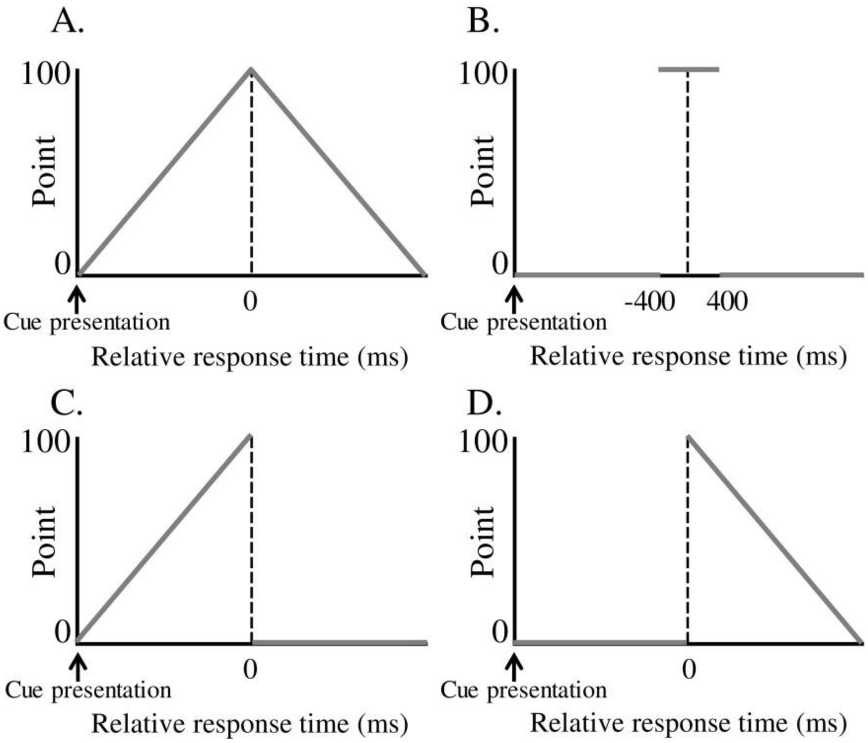

Supplementary Figure 1. The figures of gain function presented to the participants in the No Risk condition (A), the Step condition (B), the $Risk_{after}$ condition (C) and the $Risk_{before}$ condition (D). The time length from the visual cue to the target time (i.e., 2300 ms) was not informed.

1-2. Distribution of response times

We assume that participant's response times are distributed according to a Gaussian distribution for all conditions. To confirm this, we conducted Kolmogorov-Smirnov tests for each condition and participant. The results are shown in Supplementary Table 1. The null hypothesis that response time follows a Gaussian distribution was not significantly rejected for 89.2 % (33/37) of participants in the No Risk condition, for 100 % (16/16) in the $Risk_{after}$ condition, for 100 % (12/12) in the $Risk_{before}$ condition, and for 88.8 % (8/9) in the Step condition. Thus, in our coincident timing task, we can conclude that response time t follows a Gaussian distribution.

Supplementary Table 1. The results of Kolmogorov-Smirnov test in Experiment 1 (A), Experiment 2 (B), and Experiment 3 (C). Kolmogorov-Smirnov tests examine the null hypothesis that a particular parameter follows a Gaussian distribution. If this null hypothesis is rejected, the parameter does not follow a Gaussian distribution. The results show that the null hypothesis was not rejected for almost all of the participants. * indicates $p < .05$, ** indicates $p < .01$, and *** indicates $p < .001$.

(A) Experiment 1

Participants	No Risk condition				Risk$_{after}$ condition			
	D value	Degrees of freedom	*P* value		*D* value	Degrees of freedom	*P* value	
1	0.045	97	0.200		0.070	97	0.200	
2	0.055	96	0.200		0.063	100	0.200	
3	0.064	97	0.200		0.067	97	0.200	
4	0.090	99	0.045	*	0.078	98	0.159	
5	0.069	98	0.200		0.062	98	0.200	
6	0.077	97	0.182		0.055	97	0.200	
7	0.063	97	0.200		0.065	99	0.200	
8	0.038	99	0.200		0.069	97	0.200	
9	0.093	94	0.042	*	0.068	99	0.200	
10	0.055	99	0.200		0.056	100	0.200	
11	0.113	99	0.003	**	0.073	96	0.200	
12	0.098	97	0.023	*	0.075	96	0.200	
13	0.082	100	0.092		0.065	98	0.200	
14	0.058	95	0.200		0.053	98	0.200	
15	0.061	98	0.200		0.057	98	0.200	
16	0.057	95	0.200		0.073	99	0.200	

(B) Experiment 2

Participants	No Risk condition				Risk$_{before}$ condition			
	D value	Degrees of freedom	*P* value		*D* value	Degrees of freedom	*P* value	
17	0.049	98	0.200		0.068	96	0.200	
18	0.073	98	0.200		0.048	98	0.200	
19	0.078	100	0.140		0.051	97	0.200	
20	0.078	98	0.163		0.069	97	0.200	
21	0.060	95	0.200		0.074	100	0.200	
22	0.047	98	0.200		0.074	98	0.200	
23	0.068	96	0.200		0.075	100	0.188	
24	0.038	96	0.200		0.062	98	0.200	
25	0.051	97	0.200		0.049	99	0.200	
26	0.065	98	0.200		0.045	98	0.200	
27	0.065	98	0.200		0.073	96	0.200	
28	0.054	97	0.200		0.065	98	0.200	

(C) Experiment 3

Participants	No Risk condition				Step condition			
	D value	Degrees of freedom	*P* value		*D* value	Degrees of freedom	*P* value	
29	0.067	98	0.200		0.054	98	0.200	
30	0.040	97	0.200		0.067	99	0.200	
31	0.060	98	0.200		0.054	98	0.200	
32	0.059	99	0.200		0.096	98	0.027	*
33	0.045	97	0.200		0.081	99	0.104	
34	0.081	99	0.114		0.062	98	0.200	
35	0.082	98	0.100		0.074	99	0.200	
36	0.085	98	0.080		0.041	98	0.200	
37	0.049	99	0.200		0.046	99	0.200	

1-3. Magnitude of response error

We found that compensation in response to errors following miss trials was larger than that of following success trials (Figure 6). However, compensation size ($RT_{n+1} - RT_n$) should be calculated differently between conditions if the magnitude of response error on the current trial (E_n) were different in each bin. To assess this possibility, we compared response error on the current trial in the $\text{Risk}_{\text{after}}$/$\text{Risk}_{\text{before}}$ condition with that in the No Risk condition across bins. Mean response error across participants on the current trial is shown in Experiment 1 in Supplementary Figure 2A and in Experiment 2 in Supplementary Figure 2B. First, we performed two-way repeated-measures ANOVA on the response error in Experiment 2. The levels were condition (2: $\text{Risk}_{\text{before}}$ condition and No Risk condition) and bin (4: bin1-4). We found no main effect of condition (F [1, 11] = 1.76, p = 0.21) or interaction effect (F [3, 33] = 0.27, p = 0.85). Therefore, we conclude that the magnitude of the response error in the $\text{Risk}_{\text{before}}$ condition was not significantly different from that in the No Risk condition for any bin (Supplementary Figure 1B). We also performed two-way repeated-measures ANOVA on the response error in Experiment 1. The levels were condition (2: $\text{Risk}_{\text{after}}$ condition and No Risk condition) and bin (4: bin1-4). Although we found no main effect of condition (F [1, 15] = 0.00, p = 0.99), we found an interaction effect (F [2.15, 32.22] = 4.69, p < .05). The simple main effect revealed that the mean response error across participants in the $\text{Risk}_{\text{after}}$ condition was significantly smaller than that in the No Risk condition only in bin 1 (F [1, 15] = 5.02, p < .05). There was no difference between conditions in bins 2, 3, or 4 (Fs [1, 15] < 4.32, ps > .05). From these results, compensation size was calculated assuming uniform magnitudes of response error except for the case of bin 1 in Experiment 1.

Although in this bin mean response error was different between conditions, we assume that this result would have a small influence on our results on compensation size for the following reason. We focused on the compensation size following miss trials (bin 4 in Experiment 1). Mean response error was not different between conditions in this bin. Thus, a difference in the magnitude of response errors in bin 1 does not cause the observed larger compensation following miss trials.

1-4. Individual variability for the compensation size

We assume that individual variability for the compensation size is negligible. To confirm this, we showed the individual data for the compensation size in Supplementary Figure 3. The participant's samples seem to distribute normally, at least, not to distribute bimodality.

For quantitative analysis, we conducted Shapiro-Wilk tests for each bin and condition. We used Shapiro-Wilk test for this analysis due to small samples. As shown in Supplementary Table 2, the null hypothesis that distribution of the compensation size follows a Gaussian distribution was not significantly rejected for almost all of the samples. Therefore, individual variability for the compensation size is negligible and calculating average value is valid in Figure 6.

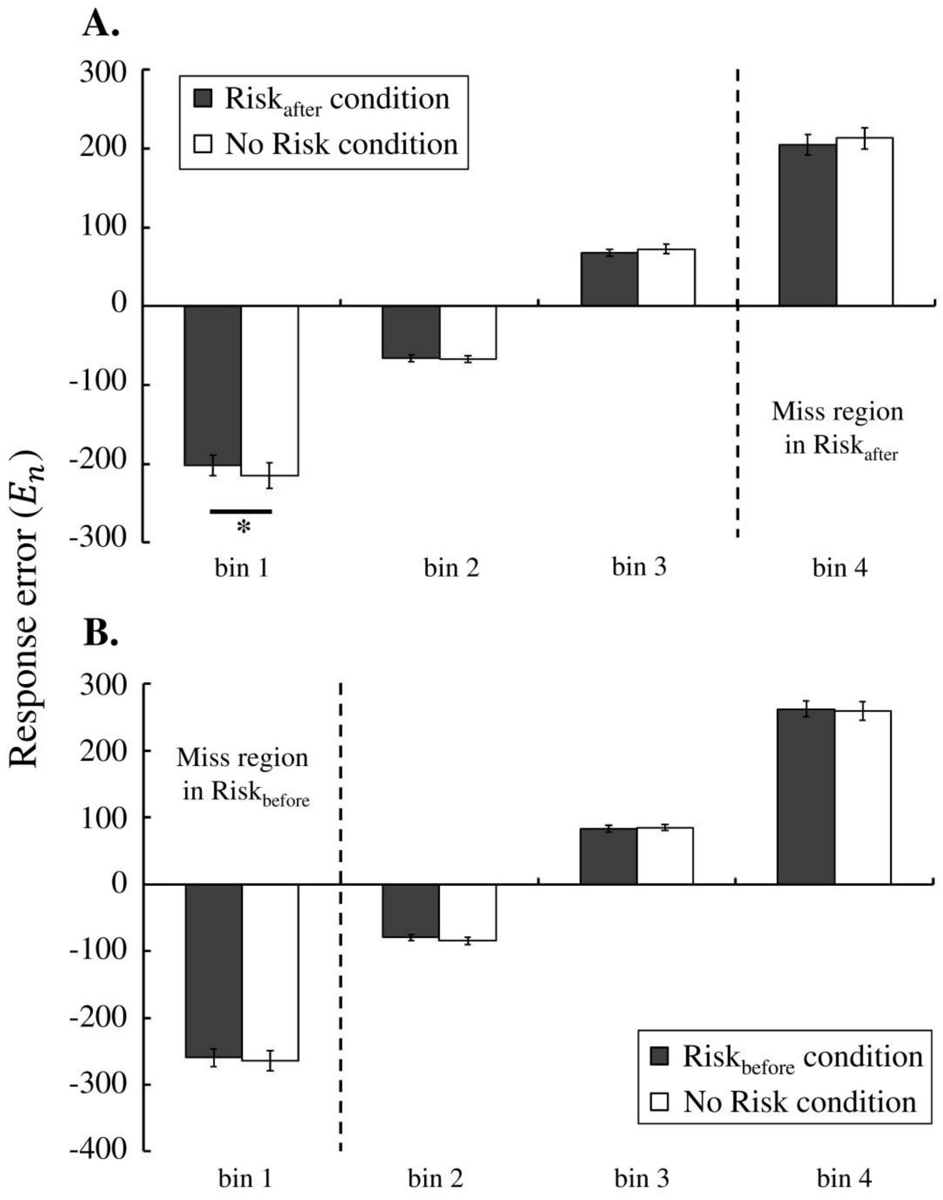

Supplementary Figure 2: The difference in mean response error on current trial between conditions in Experiment 1 (A) and Experiment 2 (B). Mean response error on current trial across participants was plotted in each bin and condition. * indicates $p < .05$. Error bars indicate standard error of the mean.

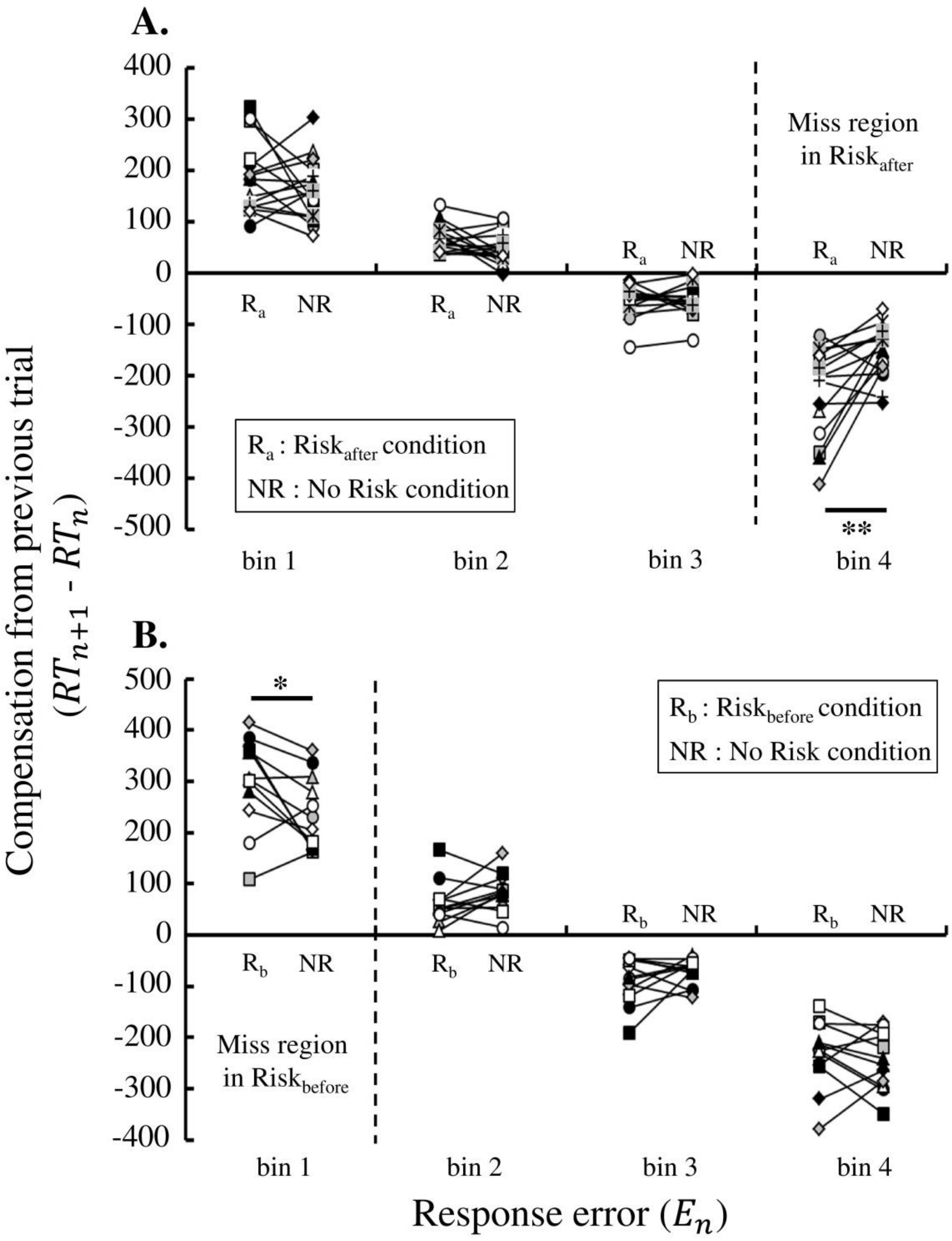

Supplementary Figure 3: Individual data for the compensation size in Experiment 1 (A) and Experiment 2 (B). Mean compensation size within one participant was plotted against the magnitude of response error for both conditions. Each colored symbol represents one participant. ** indicates $p < .01$. * indicates $p < .05$. The data averaging these participant's samples corresponds to Figure 6.

Supplementary Table 2. The results of Shapiro-Wilk test in Experiment 1 (A) and Experiment 2 (B). Shapiro-Wilk test examine the null hypothesis that a particular parameter follows a Gaussian distribution. If this null hypothesis is rejected, the parameter does not follow a Gaussian distribution. * indicates $p < .05$.

(A) Experiment 1

Bin	No Risk condition			$Risk_{after}$ condition			
	W value	Degree of freedom	*P* value	*W* value	Degree of freedom	*P* value	
1	0.965	16	0.749	0.904	16	0.093	
2	0.946	16	0.431	0.921	16	0.178	
3	0.927	16	0.222	0.864	16	0.022	*
4	0.953	16	0.533	0.906	16	0.102	

(B) Experiment 2

Bin	No Risk condition			$Risk_{before}$ condition		
	W value	Degree of freedom	*P* value	*W* value	Degree of freedom	*P* value
1	0.888	12	0.111	0.924	12	0.323
2	0.961	12	0.801	0.862	12	0.052
3	0.883	12	0.096	0.888	12	0.112
4	0.958	12	0.759	0.926	12	0.343
